# Surgical resection of a subphrenic endometrial stromal sarcoma metastasis tethered to diaphragm and liver in a 28-year-old patient: a case report

**DOI:** 10.1093/jscr/rjac494

**Published:** 2022-11-14

**Authors:** Lina Cadili, Trevor Cohen, Alison Carol Ross

**Affiliations:** Division of General Surgery, Department of Surgery, Faculty of Medicine, University of British Columbia, Vancouver, British Columbia, Canada; Division of Gynaecologic Oncology, Department of Obstetrics & Gynecology, Faculty of Medicine, University of British Columbia, Vancouver, British Columbia, Canada; Division of General Surgery, Department of Surgery, Faculty of Medicine, University of British Columbia, Vancouver, British Columbia, Canada; Department of Surgery, Vancouver Island Health Authority, Victoria, British Columbia, Canada

## Abstract

Endometrial stromal sarcomas are the second most common type of mesenchymal uterine tumors, and they represent 1% of all uterine malignancies. Metastasis of this tumor occurs in about one third of patients, usually to the pelvis and lower genital tract. Metastases to the diaphragm or liver are exceedingly rare, with only a few published cases in the literature. This case presents a 28-year-old woman with a subphrenic endometrial stromal sarcoma metastasis between the right diaphragm and segment IVa of the liver that was treated with surgical resection.

## INTRODUCTION

Endometrial stomal sarcomas (ESSs) are a rare entity representing ~1% of all uterine malignancies, and they are the second most common mesenchymal uterine tumor after leiomyosarcomas [1, 2]. The pathogenesis of this tumor is unclear, but exposure to tamoxifen, unopposed estrogens and polycystic ovarian disease are thought to be linked [3]. ESSs are typically slow-growing tumors, which present in the perimenopausal period. Recurrences develop in about one third to one half of patients and are usually limited to the pelvis and lower genital tract [3]. Due to the slow-growing nature of the tumor, distant metastasis of ESSs can present even 20 years after the initial diagnosis [3]. A theory for the cause of recurrences following oophorectomy is estrogens produced by peripheral tissues or exogenous estrogen administration (in the form of hormone replacement therapy, for example) [3].

Most published case reports are on pulmonary, cardiac and pelvic metastases of ESSs. Distant metastases of this tumor to the diaphragm or liver are extremely rare, as such the literature regarding their clinical management is scarce. Ramia *et al.* suggest liver resection in resectable patients by extrapolating data obtained on liver metastasis from other sarcomas and uterine tumors [4]. Maeda *et al.* discuss their 45-year-old patient who developed a recurrence of her low-grade ESS between the right diaphragm and liver, very similar to the case we present in this paper, treated with chemotherapy and surgical resection [5]. The National Comprehensive Cancer Network (NCCN) recommends surgical resection in uterine sarcoma isolated metastasis, with the consideration of post-operative systemic therapy or radiation therapy [6]. Here, we present a unique case of a subphrenic metastatic endometrial stromal sarcoma tethered to the right diaphragm and segment IVa of the liver in a 28-year-old woman treated with surgical resection.

## CASE REPORT

**Figure 1:**
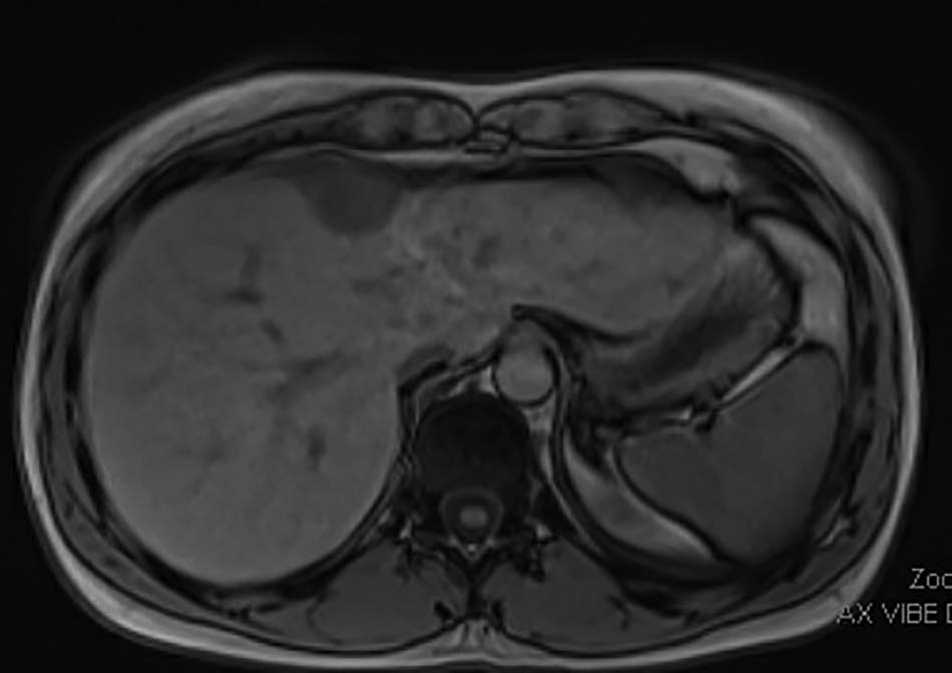
Pre-operative MRI image showing a 3.6 × 2.4 cm mass over segment IVa of the liver with possible parenchymal invasion.

The patient is a previously healthy woman who presented at the age of 21 with severe abnormal uterine bleeding. She was initially treated with uterine artery embolization, and then underwent surgical resection in the form of a radical hysterectomy, right salpingectomy, left salpingo-oophorectomy and bilateral pelvic lymph node dissection. Pathology confirmed a 9 cm low-grade endometrial stromal sarcoma with right pelvic lymph nodes positive for metastasis, consistent with stage IIIC disease. She was also treated with long-term megestrol acetate. She developed recurrence adjacent to the right ovary in 2018 (at the age of 24), which was treated with surgical resection, doxorubicin, olaratumab and pelvic radiation therapy. She did well until early 2022, at the age of 28, when a surveillance abdominal magnetic resonance imaging (MRI) scan showed a lobulated enhancing mass measuring 3.6 × 2.4 cm over segment IVa of the liver, with possible parenchymal invasion (Fig. 1). The case was discussed between a hepatopancreatobiliary surgeon and gynecologic oncologist, and surgical resection was deemed to be a reasonable treatment strategy for this tumor.

**Figure 2:**
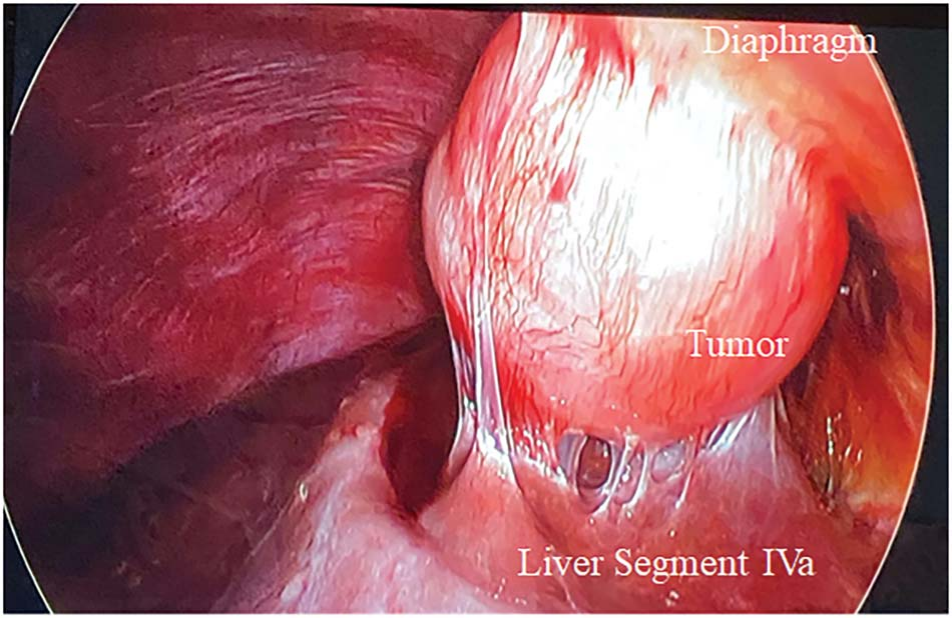
Metastatic endometrial stromal sarcoma tethered to the right diaphragm and segment IVa of the liver.

As such, the patient was brought to the operating room for a laparoscopic segment IVa liver resection. Upon insertion of the laparoscope, the mass appeared tethered to the right diaphragm and segment IVa of the liver, and not embedded in the liver parenchyma (Fig. 2). A window was made in the coronary ligament using the harmonic scalpel device. The mass was then dissected circumferentially, taking a bit of the liver capsule with each bite to ensure adequate margins. Once it was freed from the liver side, the dissection was continued at the top of the mass and a few fibers of the diaphragm were taken. A small defect was created in the diaphragm during this dissection, which was closed with metal clips. There were no complications and the patient’s post-operative course was unremarkable other than a small right pneumothorax, which did not require a chest tube. The final pathology from the tumor confirmed metastatic endometrial stromal sarcoma. Multidisciplinary discussion at the British Columbia Cancer Agency recommended active surveillance and no further treatment with megestrol acetate or letrozole necessary.

## DISCUSSION

Endometrial stromal sarcomas are rare uterine malignancies that typically present perimenopausally. They are slow-growing tumors with the potential to metastasize years after the initial diagnosis, most commonly to the pelvis and lower genital tract. Distant metastases to the diaphragm or liver are exceedingly rare; as such, the literature on this topic is limited. Our case of this young 28-year-old woman with a subphrenic ESS metastasis tethered to the right diaphragm and segment IVa of the liver is unique and highlights the importance of multidisciplinary discussion. The patient was treated with surgical resection, in keeping with the NCCN clinical guidelines for metastatic uterine sarcoma.
